# Investigation of the differences in volatile organic compounds of sesame oil under different processing methods using GC-IMS and electronic nose

**DOI:** 10.3389/fnut.2025.1611006

**Published:** 2025-08-01

**Authors:** Wen Ai, Xinyi Yin, Shijia Zhang, Ziran Yu, Yuqing Liu, Dan Huang

**Affiliations:** ^1^State Key Laboratory of Chinese Medicine Powder and Medicine Innovation in Hunan (Incubation), Academy of Chinese Medical Sciences (Science and Technology Innovation Center), Hunan University of Chinese Medicine, Changsha, China; ^2^School of Pharmacy, Hunan University of Chinese Medicine, Changsha, China

**Keywords:** sesame oil, Heracles NEO ultra-fast gas-phase electronic nose, gas chromatography–ion mobility spectrometry, volatile organic compounds, processing method

## Abstract

**Introduction:**

Sesame oil is an edible oil of high economic and nutritional value, possessing a unique flavor and exerting various physiological effects, including antioxidant, anti-inflammatory, and hypoglycemic effects. Flavor compounds are essential in evaluating the taste and quality of food. To explore the impacts of water substitution method, cold-pressing method, and hot-pressing method on the volatile organic components and active aroma components of sesame oil.

**Methods:**

This study employed the Heracles Neo ultra-fast gas-phase electronic nose and GC-IMS technology, combined with chemometric analysis, to analyze the volatile organic compounds (VOCs) of three groups of sesame oil samples.

**Results:**

A total of 74 VOCs were detected in the three sesame oil samples, which were from GC-IMS and Heracles NEO ultra-fast gas-phase electronic nose (60 VOCs were detected via GC-IMS, 22 VOCs were detected via GC-IMS, among them, 8 VOCs were simultaneously detected via GC-IMS and Heracles NEO ultra-fast gas-phase electronic nose). The sesame oil produced via the water substitution method was rich in more than 42 VOCs, including Cyclopentanone, 1-Pentanol and had a more unique and richer flavor; the sesame oil produced via the cold-pressing method contains 4 VOCs, for example, *γ* -terpinene with an original fruity flavor; and the sesame oil processed by the hot-pressing method was rich in 29 VOCs, including 2-methyl-1-propanol, and had a better fat aroma.

**Discussion:**

This study helps to improve the quality and flavor of sesame oil from the perspective of volatile components, facilitating technological innovation and industrial upgrades.

## 1 Introduction

Sesame is the seed of *Sesamum indicum* L., a major oil crop often referred to as the “queen” of oil crops. Sesame oil, derived from sesame seeds and consumed worldwide for centuries, has a unique flavor and contains a series of active ingredients such as sesamin, sesamol, and vitamin E (1, 2); these active ingredients exhibit antioxidant (3), anti-inflammatory (4), and blood-sugar-lowering (5) properties, among other physiological benefits.

Volatile organic compounds (VOCs) significantly influence the flavor of products. Current research on VOCs in sesame oil mainly focuses on the types and contents of VOCs and the effects of different processing technologies on the flavor of sesame oil (6–8). The major active substances in sesame oil include pyrazines, furans, thiazoles, thiophenes, and pyrroles, as well as alcohols, aldehydes, ketones, acids, and esters (9), which greatly contribute to the overall aroma of sesame oil.

The commonly used methods for processing sesame oil are pressing method, leaching method, and water substitution method. Pressing, one of the most widely used techniques, employs mechanical force to rupture sesame cells, thereby liberating oil. Depending on the operational temperature, pressing can be classified as either cold or hot. Cold pressing, conducted at lower temperatures, minimizes the oxidation and decomposition of unsaturated fatty acids, thus preserving the oil’s natural flavor by preventing the volatilization and deterioration of aromatic compounds. In contrast, while hot pressing disrupts the cellular structure and increases membrane permeability to facilitate greater oil extraction, it may result in the loss of VOCs and promote oxidation and polymerization reactions that alter the oil’s flavor. The leaching method, which is based on the principle of similar solubility, involves the penetration of organic solvent molecules into sesame cell gaps, where they interact with oil molecules to extract the oil. Although this method yields a high oil recovery at lower production costs, it may leave residual chemical contaminants. The water substitution method, a traditional technique, exploits the hydrophobic differences between sesame oil and the hydrophilic components (such as proteins and sugars) present in the seeds. By stirring and shaking with water, the oils are separated from sesame paste, but results in a low yield. However, the sesame oil obtained is more popular with consumers due to its mellower and longer-lasting taste and unique, rich flavor.

The electronic nose, equipped with multiple chemical sensors to emulate human olfaction, enables the efficient and accurate detection of VOCs and provides a scientific, objective means of analyzing aroma substances. The widespread application of this technology has invigorated quality control and scientific research in food and environmental fields (10–12). Gas chromatography–ion mobility spectrometry (GC-IMS) can gradually separate complex volatile aroma substances and conduct qualitative analysis of VOCs by assessing migration time of substances and using its own database. Using this method for VOCs detection offers high sensitivity, rapid analysis, real-time detection, high selectivity and a wide range of applications. These advantages make GC-IMS technology an important analytical tool in many fields (13–16).

Currently, there is a notable lack of comprehensive and systematic comparative analyses on the composition of sesame oil produced from the same batch of sesame seeds using three distinct processes: cold pressing, hot pressing, and water substitution. This study combined electronic nose and GC-IMS technologies with chemometrics to conduct a comparative analysis of the VOCs in sesame oil extracted using the water substitution method, cold-pressing method, and hot-pressing method. The results not only reveal the influence of different processing methods on the flavor of sesame oil, but also provide a scientific basis for quality control, process optimization and new product development. This, in turn, can improve the market value of products and consumer trust.

## 2 Materials and methods

### 2.1 Materials

The sesame seeds were collected from Chaoyang, Liaoning province, China (located at 102.065°E, 41.423°N) dried in the sun, the moisture content is 6%, The sesame seeds were packed and stored at 4°C until used.

### 2.2 Sample preparation

The extraction of sesame oil via the water substitution method was performed as follows: First, 200 g of sesame seeds were fried in a pan at 120°C until brown and ground with a stone mill. Hot water (100°C) was then added in a ratio of 1:1 (w/v) and stirred at 350 rpm for 30 min using a magnetic stirrer (DF-101S, Gongyi Yuhua Instrument CO., LTD, Tangshan, China), after which it was placed in a beaker and shaken for 4 h using constant temperature oscillator (Jintan Jincheng Guosheng Experimental Instrument Factory, Jiangsu, China). The upper layer of the oil was then placed in a refrigerator at 4°C for 24 h. Centrifugal filtration (6,000 rpm, 5 min) was used to obtain the sesame oil, which was named SS-01.

The extraction of sesame oil via the cold-pressing method was performed as follows: First, 200 g of sesame seeds was placed in a press oil machine (Bestday ZYJ-9029, Jiangmen, Guangdong, China) in cold-pressed mode (pressure up to 1,600 kN, 40–60°C) for pressing and filtering. Centrifugal filtration (6,000 rpm, 5 min) was then performed to obtain sesame oil, which was placed in a refrigerator at 4°C for later use. This oil was named SS-02.

The extraction of sesame oil via the hot-pressing method was performed as follows: First, 200 g of sesame seeds was placed in a press oil machine (Bestday ZYJ-9029, Jiangmen, Guangdong, China) in hot-pressing mode (frying for 20 min, pressure up to 1,600 kN, about 130°C), pressed and filtered, and centrifuged to obtain sesame oil (6,000 rpm, 5 min). This oil was then placed in a refrigerator at 4°C for later use and named SS-03.

### 2.3 GC-IMS analysis

Based on He et al. (17), but with adjustments, a FlavorSpec® gas-phase ion mobility spectrometer from G. A. S. (Dortmund, Germany) and MXT-wax capillary column (15 m × 0.53 mm, 1.0 μm) (Restek, United States) were used for the analysis of VOCs in sesame oil. IMS detector conditions as fellow: IMS detector: FlavourSpec® Gas-Phase Ion Mobility Spectrometer, G. A. S. (Dortmund, Germany); Ionization source: tritium source (3H); migration tube: 53 mm; electric field strength: 500 V/cm; migration tube temperature: 45°C; drift gas: N_2_ (purity ≥ 99.999%); flow rate: 150 mL/min; positive ion mode.

Six ketones (2-butanone, 2-pentanone, 2-hexanone, 2-heptanone, 2-octanone, and 2-nonanone) were detected, and a calibration curve of retention time and retention index was established. First, 1 mL of sesame oil was transferred into a 20 mL headspace bottle. The headspace bottle was heated to 80°C and incubated for 15 min. Then, 200 μL of the sesame oil was injected into the instrument in non-shunt mode. The running time was 50 min and the flow rate was initially 2.0 mL/min; this was linearly increased to 100 mL/min within 18 min and held for 30 min. Each sample was measured in three parallel groups.

### 2.4 Heracles NEO ultra-fast gas-phase electronic nose analysis

Optimized conditions for the detection of sesame oil using the Heracles NEO Ultra-Fast Gas-Phase electronic nose were established by refining the detection parameters used in a previous study. The specific parameters included the following: Heracles NEO ultra-fast gas-phase electronic nose, equipped with PAL RSI fully automatic headspace sampler, non-polar chromatography column MXT-5, medium Polar chromatography column MXT-1701 (Alpha MOS company, France), a sample bottle size of 20 mL; a sesame oil quantity of 5 g; an incubation temperature of 80°C; an incubation time of 20 min; an initial temperature of 30°C; a final temperature of 240°C; a capture duration of 45 s; an inlet temperature of 200°C; an injection volume of 5,000 μL; an injection speed of 250 μL/s; and an injection duration of 40 s. The initial column temperature was 40°C, with a heating mode of 1.0°C/s to 80°C and 1.5°C/s to 250°C. The acquisition time was 190 s, and the detector temperature was 260°C. The compounds were analyzed using the AroChemBase database (2021 version, Alpha MOS Corporation, Toulouse, France). Each sample was measured in five parallel groups.

### 2.5 Statistical analysis

Several plugins were used to analyze VOCs in VOCal data processing software (from G. A. S., Dortmund, Germany, version 2.0.0), including Reporter and Gallery Plot. These tools focused on 3D spectra, 2D spectra, and fingerprints. Principal component analysis (PCA) was conducted using OmicShare Tools (18), while partial least-squares regression analysis (PLS-DA) was performed using TBtools and SIMCA (Version 14.1, Umetrics, Sweden). One-way ANOVA using GraphPad Prism 8.3 (GraphPad Software, Boston, United States).

## 3 Results and discussion

### 3.1 Analysis of GC-IMS results of sesame oil samples using different processing methods

#### 3.1.1 Comparison of VOCs in sesame oil processed via different methods

**Figure 1:**
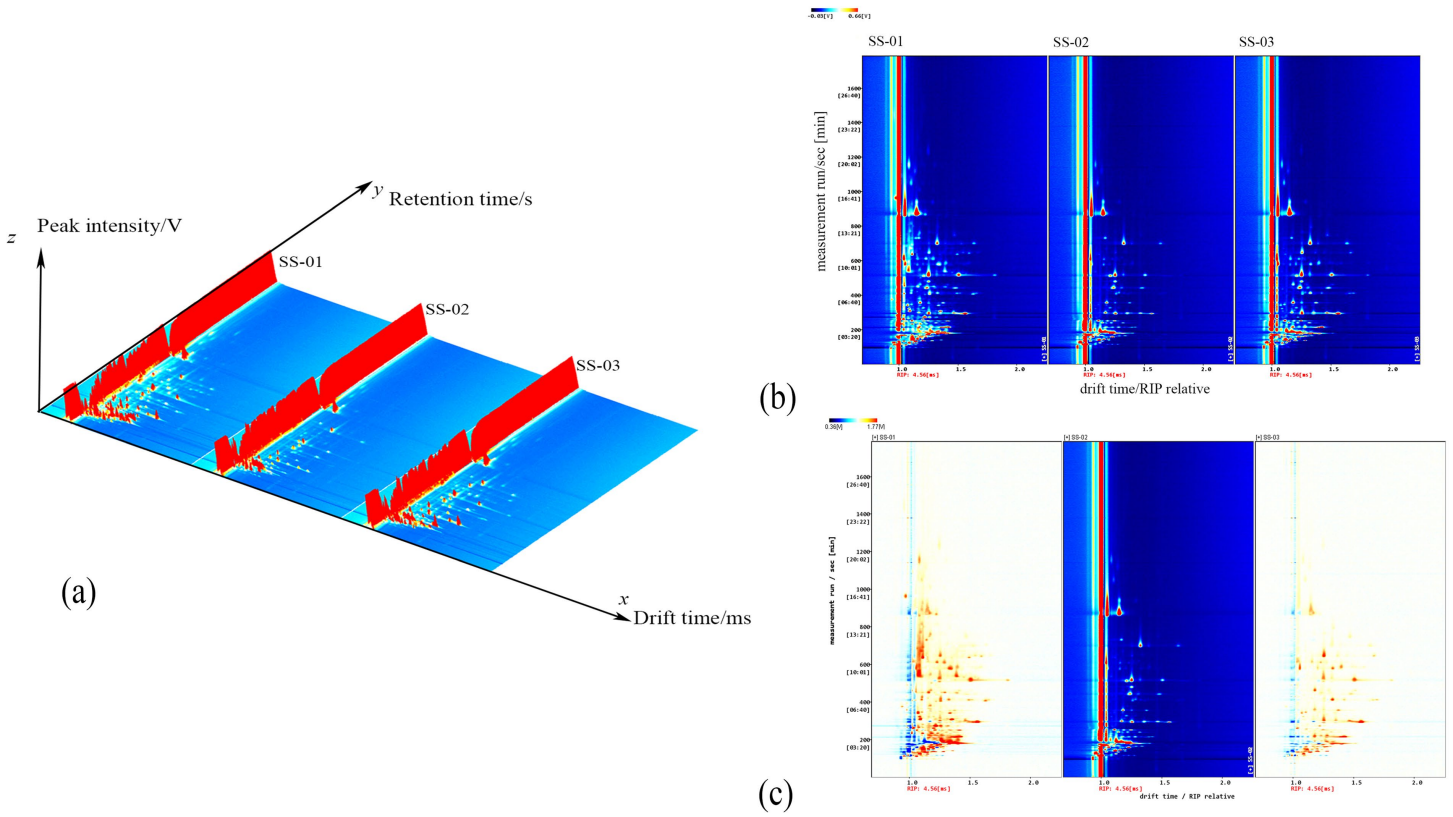
**(a)** Three-dimensional spectrum of VOCs of three groups of sesame oil; **(b)** Two-dimensional spectrum of VOCs of three groups of sesame oil; **(c)** Spectral comparison of cold-pressing method and the other two groups of sesame oil.

Figure 1a displays the three-dimensional spectra of VOCs in sesame oil obtained using different extraction methods. Each peak represents a volatile component, and the height of the red protrusion indicates the content of the respective component. As can be seen from Figure 1a, there are discernible differences in the VOC profiles of the sesame oils depending on the processing method employed.

The two-dimensional top view of the gas-phase ion migration spectrum of the VOCs in sesame oil processed via different methods is shown in Figure 1b. The x axis represents the ion migration time and the y axis represents the retention time. The red vertical line in Figure 1b represents the reaction ion peak, with the bright spots on either side indicating volatile components. The color and size of the bright spot represent the content of the volatile component, with darker colors and larger areas indicating higher contents. Red represents higher contents, while white represents lower contents. This chart enables a visual comparison of the differences in the volatile components of sesame oil samples after different processing methods.

To visually compare the differences in VOCs of sesame oil processed via different methods, the cold-pressed method spectrum was chosen as the reference. Furthermore, the reference spectrum was subtracted from the spectra of other samples to generate a comparison diagram illustrating the differences between samples processed via different methods, as shown in Figure 1c. If the volatile organic compound content in the target sample and the reference is the same, the background is white after subtraction. In addition, red indicates that the concentration of the substance in the target sample is higher than that in the reference, and blue indicates that the concentration of the substance in the target sample is lower than that in the reference.

#### 3.1.2 Analysis of VOCs in sesame oil via GC–IMS

Sixty VOCs were detected in the three sesame oil samples via GC-IMS; including 16 aldehyde compounds (26.7%); 16 alcohol compounds (26.7%); 14 ketone compounds (23.3%); and 5 pyrazine compounds (8.3%). Additionally, furans, terpenes, thiazoles, pyrroles, esters and acid compounds were detected. The results are summarized in Table 1.

**Table 1:** Comparative analysis of the detected VOCs based on RI, Rt, Dt and peak area in sesame oil.

No	Compound	CAS	Molecular Formula	MW	RI	Rt/s	Dt/ms	Peak area(mean±SD)		
								SS-01	SS-02	SS-03
1	γ-Butyrolactone	96–48-0	C_4_H_6_O_2_	86.1	1572.3	1148.386	1.08886	674.68 ± 22.06	269.01 ± 18.44	351.16 ± 14.75
2	Propanoic acid	79–09-4	C_3_H_6_O_2_	74.1	1535.4	1049.483	1.10476	319.39 ± 9.52	215.16 ± 9.82	292.55 ± 13.66
3	Acetic acid-M	64–19-7	C_2_H_4_O_2_	60.1	1455.2	862.753	1.05324	6354.96 ± 19.84	6958.3 ± 58.28	6916.01 ± 28.78
4	Acetic acid-D	64–19-7	C_2_H_4_O_2_	60.1	1458.7	870.336	1.15472	9377.37 ± 376.35	6773.91 ± 198.17	8941.19 ± 182.17
5	3-(methylsulfanyl)propanal	3,268-49-3	C_4_H_8_OS	104.2	1459.7	872.322	1.08838	581.35 ± 14.36	431.71 ± 16.86	495.94 ± 10.82
6	2,3-dimethyl-5-ethylpyrazine	15,707–34-3	C_8_H_12_N_2_	136.2	1457.4	867.469	1.22866	158.15 ± 4.03	37.99 ± 2.19	57.96 ± 1.4
7	2-ethyl-5-methylpyrazine	13,360–64-0	C_7_H_10_N_2_	122.2	1402.8	759.254	1.17605	121.22 ± 6.06	20.52 ± 0.91	27.14 ± 2.42
8	2-ethyl-6-methylpyrazine	13,925–03-6	C_7_H_10_N_2_	122.2	1387.4	731.280	1.16993	69.27 ± 0.31	69.04 ± 4.48	62.83 ± 1.02
9	2,3,5- trimethylpyrazine	14,667–55-1	C_7_H_10_N_2_	122.2	1397.2	748.959	1.17083	38.72 ± 2.59	13.35 ± 0.44	13.49 ± 1.47
10	2,4,5-trimethylthiazole	13,623–11-5	C_6_H_9_NS	127.2	1387.2	730.908	1.14584	125.08 ± 11.66	40.48 ± 4.3	37.05 ± 1.04
11	1 H-pyrrole	109–97-7	C_4_H_5_N	67.1	1499.0	960.155	0.97414	514.79 ± 2.53	125.35 ± 12.08	132.54 ± 6.89
12	1-hexanol-M	111–27-3	C_6_H_14_O	102.2	1369.1	699.304	1.32710	1170.76 ± 25.83	1121.9 ± 7.97	1359.68 ± 5.91
13	1-hexanol-D	111–27-3	C_6_H_14_O	102.2	1368.9	699.034	1.63805	250.42 ± 15.57	196.96 ± 11.33	313.6 ± 13.47
14	(E)-2-Heptenal-M	18,829–55-5	C_7_H_12_O	112.2	1336.7	646.035	1.25764	647.6 ± 12.32	182.55 ± 20.08	1202.36 ± 2.13
15	(E)-2-Heptenal-D	18,829–55-5	C_7_H_12_O	112.2	1335.9	644.821	1.66983	56.41 ± 2.72	25.41 ± 4.31	137.08 ± 5.42
16	1-Hydroxy-2-propanone	116–09-6	C_3_H_6_O_2_	74.1	1313.6	610.642	1.23151	246.85 ± 47.82	84.34 ± 76.04	117.09 ± 20.56
17	Cyclohexanone	108–94-1	C_6_H_10_O	98.1	1290.3	575.552	1.15987	125.99 ± 8.2	126.8 ± 2.02	122.44 ± 8.24
18	3-Hydroxy-2-butanone	513–86-0	C_4_H_8_O_2_	88.1	1291.7	578.117	1.33252	613.16 ± 51.08	74.94 ± 17.5	360.03 ± 23.65
19	1-octanal	124–13-0	C_8_H_16_O	128.2	1295.7	584.561	1.40030	326.11 ± 6.08	83.39 ± 6.33	381.36 ± 4.02
20	γ-Terpinene	99–85-4	C_10_H_16_	136.2	1252.1	511.297	1.21107	436.47 ± 4.73	595.32 ± 10.19	534.15 ± 9.9
21	1-Pentanol-M	71–41-0	C_5_H_12_O	88.1	1251.7	510.695	1.25176	450.72 ± 11.72	546.64 ± 23.66	598.24 ± 8.22
22	1-Pentanol-D	71–41-0	C_5_H_12_O	88.1	1254.0	514.234	1.50948	220.96 ± 6.08	281.99 ± 11.27	459.87 ± 5.76
23	3-Octanone	106–68-3	C_8_H_16_O	128.2	1256.6	518.452	1.32498	252.8 ± 23.08	218.48 ± 16.51	337.66 ± 12.86
24	(E)-2-hexenal	6,728-26-3	C_6_H_10_O	98.1	1220.0	462.764	1.18334	215.09 ± 7.92	57.62 ± 1.69	345.15 ± 7.21
25	2-pentyl furan	3,777-69-3	C_9_H_14_O	138.2	1231.8	480.095	1.25426	490.45 ± 7.67	432.18 ± 6.67	497.39 ± 4.93
26	2-Methyl-1-butanol-M	137–32-6	C_5_H_12_O	88.1	1203.9	440.302	1.23751	1192.91 ± 28.45	1105.14 ± 16.22	1363.31 ± 13.85
27	2-Methyl-1-butanol-D	137–32-6	C_5_H_12_O	88.1	1205.7	442.651	1.47746	418.53 ± 1.6	304.64 ± 9.3	494.36 ± 6.54
28	2-Heptanone-M	110–43-0	C_7_H_14_O	114.2	1179.5	407.785	1.26202	610.98 ± 11.34	202.37 ± 5.58	313.76 ± 4.37
29	2-Heptanone-D	110–43-0	C_7_H_14_O	114.2	1178.6	406.597	1.63245	192.37 ± 5.48	24.33 ± 1.38	65.89 ± 4.24
30	Heptaldehyde	111–71-7	C_7_H_14_O	114.2	1182.2	411.551	1.32887	259.34 ± 8.44	58.63 ± 3.22	447.01 ± 5.68
31	1-butanol-M	71–36-3	C_4_H_10_O	74.1	1139.1	353.953	1.1829	795.01 ± 8.03	612.74 ± 4.54	904.38 ± 6.91
32	1-butanol-D	71–36-3	C_4_H_10_O	74.1	1141.0	356.223	1.37698	259.08 ± 7.34	140.53 ± 4.61	347.97 ± 8.02
33	3-Penten-2-one-M	625–33-2	C_5_H_8_O	84.1	1129.2	341.787	1.07845	497.72 ± 7.11	48.24 ± 2.87	182.55 ± 3.6
34	3-Penten-2-one-D	625–33-2	C_5_H_8_O	84.1	1128.4	340.896	1.34143	172.08 ± 2.61	9.66 ± 0.77	34.57 ± 3.62
35	(E)-2-Pentenal	1,576–87-0	C_5_H_8_O	84.1	1131.9	345.024	1.11150	142.49 ± 3.84	43.88 ± 3.48	151.91 ± 5.15
36	2-Pentanol	6,032-29-7	C_5_H_12_O	88.1	1116.2	326.534	1.21159	121.51 ± 5.16	95.01 ± 3.9	119.79 ± 0.96
37	2-Methyl-1-propanol-M	78–83-1	C_4_H_10_O	74.1	1089.5	297.277	1.17006	295.16 ± 6.9	506.16 ± 7.65	359.05 ± 9.73
38	hexanal-M	66–25-1	C_6_H_12_O	100.2	1083.5	291.523	1.25813	1501.76 ± 27.09	1088.02 ± 6.03	1839.76 ± 14.88
39	hexanal-D	66–25-1	C_6_H_12_O	100.2	1083.9	291.935	1.56339	1979.28 ± 24.38	511.48 ± 6.01	2140.02 ± 10.5
40	2-Methyl-1-propanol-D	78–83-1	C_4_H_10_O	74.1	1089.3	297.086	1.35403	162.96 ± 4.2	205.46 ± 6.52	215.87 ± 3.87
41	2,3-pentadione	600–14-6	C_5_H_8_O_2_	100.1	1060.1	270.869	1.22073	1345.07 ± 18.01	21.71 ± 6.05	103.92 ± 1.31
42	1-propanol-M	71–23-8	C_3_H_8_O	60.1	1034.4	249.829	1.11429	3564.22 ± 28.84	2152.01 ± 77.2	3306.81 ± 18.69
43	1-propanol-D	71–23-8	C_3_H_8_O	60.1	1036.7	251.648	1.25047	1188.5 ± 11.18	366 ± 21.58	975.61 ± 8.11
44	2-butanol-M	78–92-2	C_4_H_10_O	74.1	1020.8	239.320	1.14521	103.57 ± 3.62	174.47 ± 2.92	182.1 ± 3.19
45	2-butanol-D	78–92-2	C_4_H_10_O	74.1	1021.7	239.977	1.32021	158.96 ± 3.09	118.35 ± 2.7	179.88 ± 1.91
46	n-Pentanal-M	110–62-3	C_5_H_10_O	86.1	985.3	214.042	1.17535	301.6 ± 10.13	207.29 ± 3	434.34 ± 6.21
47	n-Pentanal-D	110–62-3	C_5_H_10_O	86.1	986.7	214.968	1.42397	244.1 ± 6.22	39.9 ± 3.01	375.73 ± 2.83
48	2-Pentanone	107–87-9	C_5_H_10_O	86.1	986.5	214.812	1.37195	283.37 ± 8.54	230.74 ± 11.18	226.24 ± 10.5
49	2-Methylbutanal	96–17-3	C_5_H_10_O	86.1	927.7	178.259	1.40216	820.09 ± 14.51	89.84 ± 2.33	465.97 ± 5.03
50	Acetic acid ethyl ester	141–78-6	C_4_H_8_O_2_	88.1	903.7	165.153	1.34063	70.13 ± 3.24	73.93 ± 4.66	83.3 ± 2.13
51	2-Methyl propanal	78–84-2	C_4_H_8_O	72.1	846.3	137.645	1.28483	163.06 ± 3.18	792.04 ± 4.6	555.79 ± 4.92
52	Butanal	123–72-8	C_4_H_8_O	72.1	899.0	162.750	1.28306	200.92 ± 3.05	94.35 ± 6.16	221.55 ± 4.24
53	2-propanone	67–64-1	C_3_H_6_O	58.1	814.6	124.461	1.12522	268.53 ± 3.6	26.06 ± 2.3	60.59 ± 1.62
54	Propanal	123–38-6	C_3_H_6_O	58.1	810.3	122.767	1.08990	1157.42 ± 37.59	150.45 ± 10.17	615.61 ± 25.69
55	1-Penten-3-ol	616–25-1	C_5_H_10_O	86.1	1157.1	377.036	0.94494	298.19 ± 1.72	114.08 ± 3.12	173.73 ± 3.84
56	2-Butanone	78–93-3	C_4_H_8_O	72.1	917.6	172.624	1.24939	1368.08 ± 19.37	92.37 ± 8.07	237.72 ± 6.13
57	2-methyl-2-propenal	78–85-3	C_4_H_6_O	70.1	887.0	156.627	1.06342	239.97 ± 4.27	6.4 ± 0.5	148.1 ± 1.86
58	2,5-Dimethylpyrazine	123–32-0	C_6_H_8_N_2_	108.1	1329.3	634.546	1.11673	504.78 ± 12.05	44.88 ± 9.18	49.68 ± 1.54
59	Cyclopentanone	120–92-3	C_5_H_8_O	84.1	1182.9	412.493	1.10910	200.5 ± 3.61	20.32 ± 1.61	26.62 ± 1.63
60	1-Penten-3-one	1,629-58-9	C_5_H_8_O	84.1	1055.1	266.613	1.08373	142.95 ± 2.42	28.75 ± 3.64	37.85 ± 0.61

The substance suffixes M and D represent monomers and dimers of the same substance, respectively, RI is the retention index, Rt is the retention time, Dt is the drift time, SD is standard deviation, Peak area is presented as means ± SD (*n* = 3).

#### 3.1.3 GC-IMS fingerprint analysis of VOCs in sesame oils

**Figure 2:**
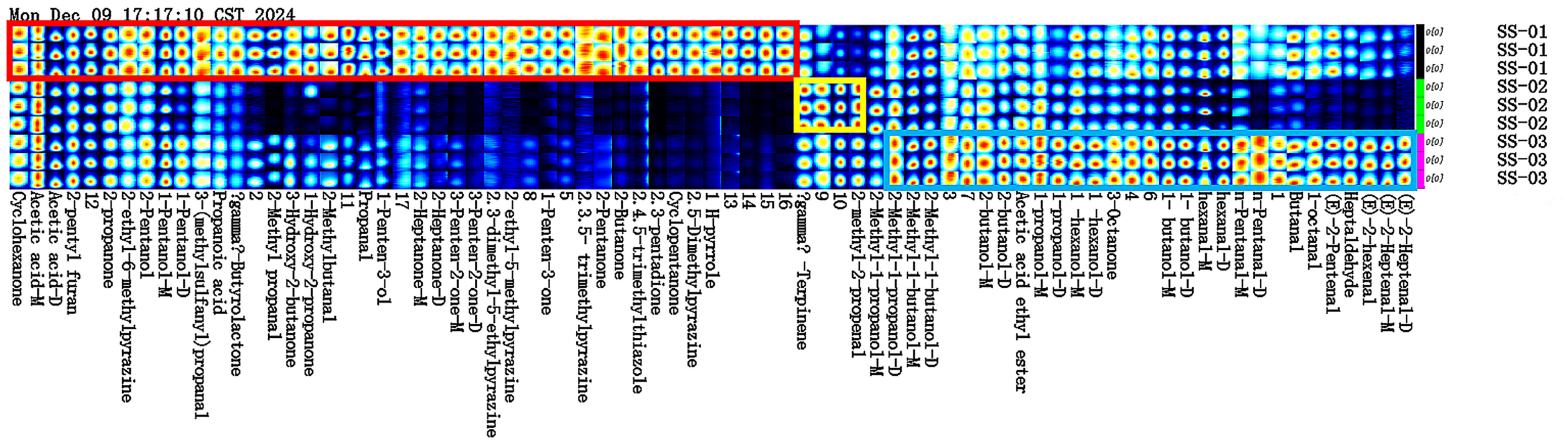
Fingerprint analysis of VOCs in sesame oils. (The red box represents the compounds with the SS-01 sample higher than the other two groups, the yellow box represents the compounds with the SS-02 sample higher than the other two groups, and the blue box represents the compounds with the SS-03 sample higher than the other two groups).

Figure 2 shows the differences in VOCs among the three groups of samples. As can be seen from the figure: acetic acid, 2-pentyl furan, 2-propanone and many other 28 compounds and other substances were more abundant in the SS-01 sample. The SS-02 sample exhibited high contents of *γ*-Terpinene and 2-methyl-2-propenal. 2-Methyl-1-propanol, 2-Methyl-1-butanol, 2-butanol and other 13 compounds were high levels in the SS-03 sample. Through the comparative analysis of fingerprints, we can find that the number of VOCs obtained by the extraction of sesame oil via the water substitution method was the largest and the variety was abundant, while the VOCs obtained by the extraction of sesame oil via the cold-pressing method were the least abundant and single, so we can easily know that the VOCs of sesame oil treated by different processing methods are very different.

#### 3.1.4 Principal component analysis

A PCA score graph was created through dimensionality reduction and the linear transformation of the sample’s original data. The distance between samples in the graph illustrates their differences; closer distances indicate smaller differences, while farther distances represent greater differences (19).

**Figure 3:**
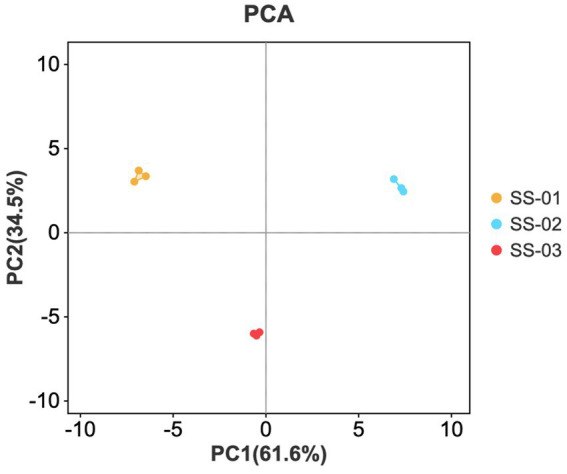
PCA scores of VOCs in the three groups of sesame oils.

PCA dimensionality reduction was utilized to study differences in sesame oil flavor processing via various methods. This study found a cumulative contribution rate of 96.1% for the principal components, with PC1 and PC2 contributing 61.6 and 34.5%, respectively. Figure 3 showed the parallel samples clustered closely and showed good parallelism, while the separation between samples was higher and the differences between groups more apparent. The differences between samples align with the intuitive observation results obtained for the fingerprint spectrum.

#### 3.1.5 Partial least-squares discriminant analysis

**Figure 4:**
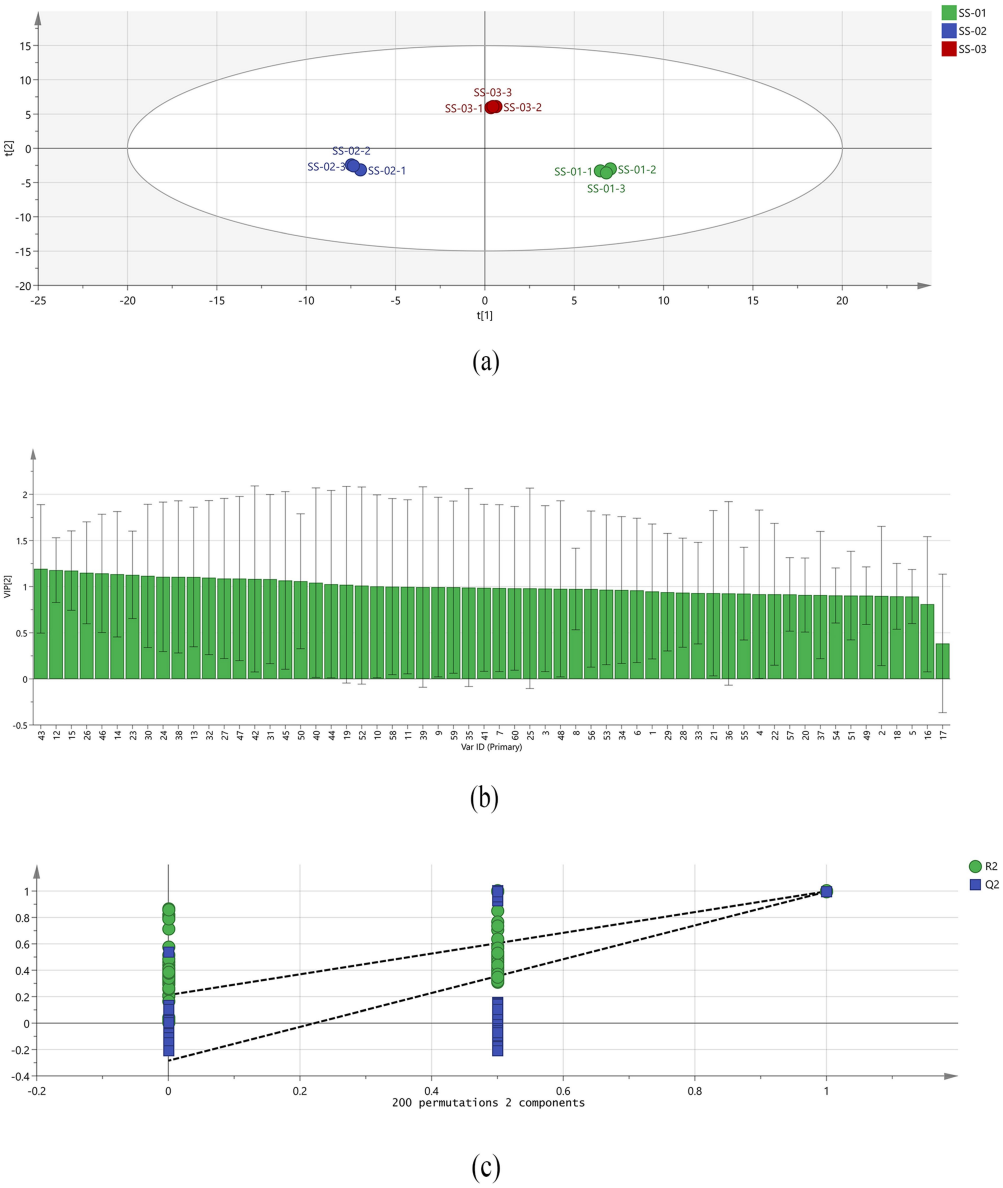
**(a)** PLS − DA analysis of VOCs in the three groups of sesame oil; **(b)** VIP values of the characteristic variables; **(c)** Permutation test results for VOCs in the three groups of sesame oil.

PLS-DA is a supervised discriminant modelling method that effectively explains the observed values and predicts the corresponding variables (20). The model’s reliability and predictive capacity are evaluated using *R*^2^ and *Q*^2^; values above 0.5 indicate an acceptable fit, with values closer to 1 indicating a stronger predictive capacity. The model was used to import data from the three sample groups processed via different methods. The results, depicted in Figure 4a, show *R*^2^X = 0.961, *R*^2^Y = 0.998, *Q*^2^ = 0.996.

In addition, to measure the contribution of each variable, projection importance (VIP) of each volatile component variable was predicted based on the PLS-DA model. When VIP is greater than 1, the variable significantly contributes to the overall discriminant model. As shown in Figure 4b, 1-propanol-D, 1-hexanol-M, (E)-2-Heptenal-D, 2-Methyl-1-butanol-M, n-Pentanal-M, (E)-2-Heptenal-M, 3-Octanone, Heptaldehyde, (E)-2-hexenal, hexanal-M, 1-hexanol-D, 1-butanol, 2-Methyl-1-butanol-D, n-Pentanal-D, 1-propanol-M, 1-butanol-M, 2-butanol-D, Acetic acid ethyl ester, 2-Methyl-1-propanol-D, 2-butanol-M, 1-octanal, Butanal and 2, 4, 5-trimethylthiazole are the main components that indicate a difference. These compounds play an important role in distinguishing between sesame oil samples that have been treated using different processing methods, and represent the main marker compounds. To determine whether the model was overfitted, 200 cross-validations were conducted simultaneously to examine the R^2^ and Q^2^ values. In the permutation test, Q^2^ drops sharply from 0.996 to −0.285, and R^2^Y decreases sharply. Meanwhile, the large slope in the Figure 4c indicates the PLS-DA model was not overfitting (*R*^2^ = 0.212, Q^2^ = −0.285, as shown in Figure 4c).

### 3.2 Analysis of electronic nose results on VOCs of sesame oil using different processing methods

#### 3.2.1 Gas chromatogram analysis

**Figure 5:**
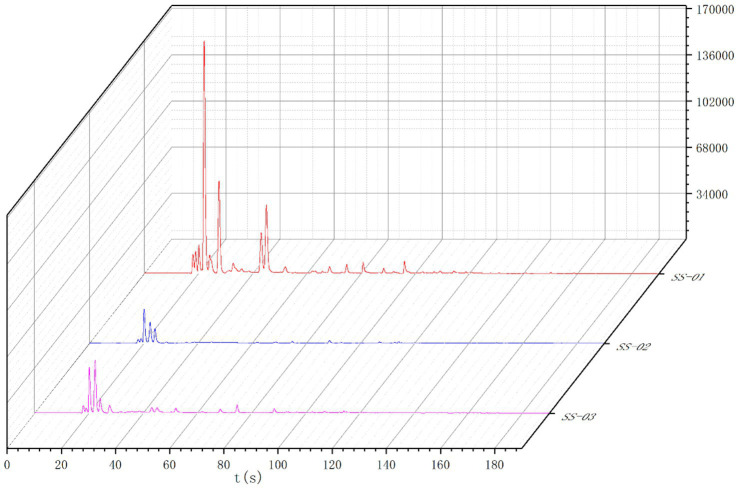
MXT-5 gas chromatogram overlay diagram.

**Figure 6:**
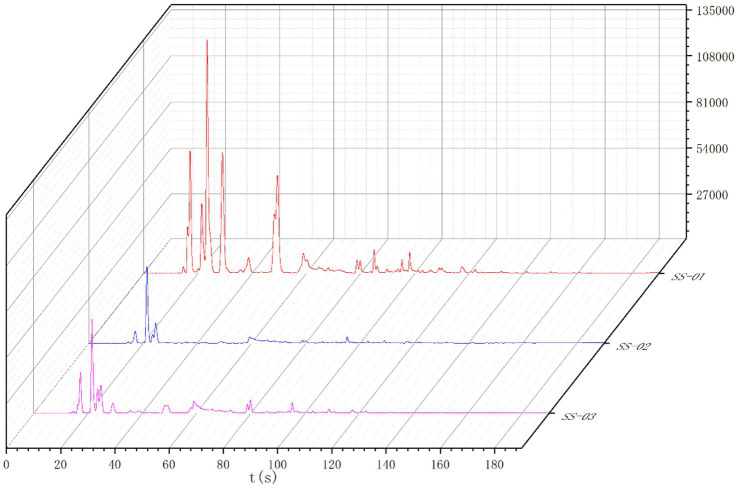
MXT-1701 gas chromatogram overlay diagram.

The Heracles NEO ultra-fast gas-phase electronic nose has two ionization detectors, namely the MXT-5 and the MXT-1701 chromatography columns. This study utilized both detectors to compare the differences between samples more accurately. The results are shown in Figures 5, 6. Analysis of the detection results using the chromatographic column showed generally similar results between the two columns, with differences in retention time and peak area between three sesame oil samples. The red SS-01 sample had a higher peak than the other two samples between 0 and 60 s, and a characteristic peak near 20 s. The pink SS-03 sample and the blue SS-02 sample exhibited less significant differences in peak height between 80 s and 180 s, but the red SS-01 sample had higher peaks at different times. The difference between the samples was mainly evidenced by the change in peak height, or the number of volatile components. Further differences between the sample groups were determined using PCA statistics and the qualitative identification of differential volatile odor substances. This process helped to accurately and effectively determined the differences in the volatile components of sesame oil processed using different methods.

#### 3.2.2 Principal component analysis (PCA)

**Figure 7:**
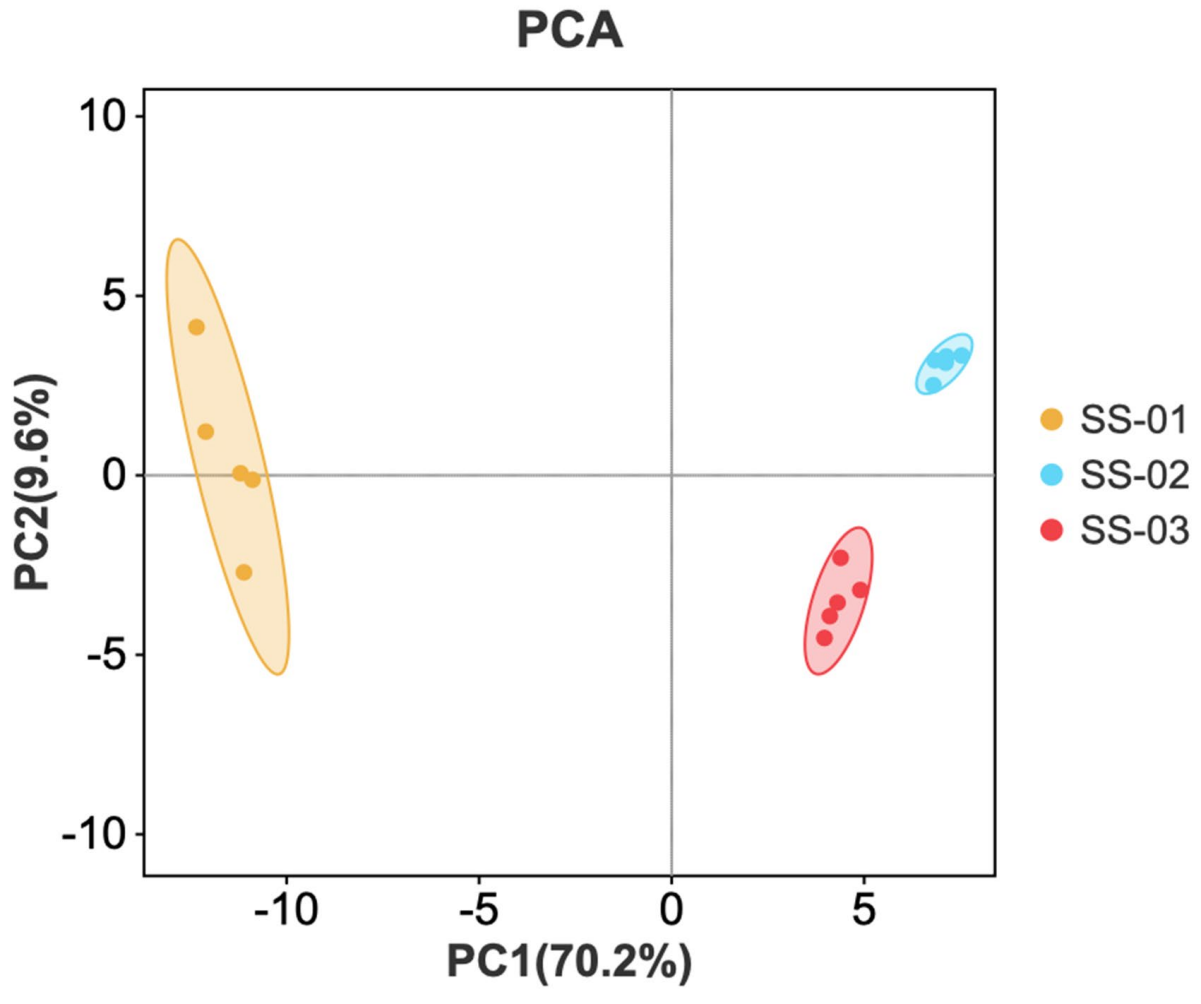
PCA scores of VOCs in the three groups of sesame oils.

PCA was performed on the data of the three groups of sesame oil samples to obtain the principal component analysis diagram, as shown in Figure 7. The horizontal and vertical axes represent the contribution rates of the first and second principal components obtained via PCA. The contribution rate of the first principal component is 70.2%, and the second is 9.6%. In the diagram, smaller distances indicate smaller sample differences, while larger distances indicate greater differences. The positions of SS-02 and SS-03 are similar, indicating a small odor difference between the two groups. SS-01 is located alone on the left side of the area and is the sample with the largest odor difference.

#### 3.2.3 Qualitative identification of different compounds

The AroChemBase database was utilized to analyze the chromatographic peaks of the three sesame oil samples. The analysis provides possible compounds and sensory descriptions for each sample. Tables 2 show the qualitative results, with “odor threshold” indicating the minimum concentration at which a specific odor could be perceived, and lower thresholds indicating stronger odors. The average peak area of the sesame oil samples at different retention times is also shown in these tables, with the peak area representing the content of the compound. A higher content corresponds to larger average peak areas.

**Table 2:** Differential chromatographic peak qualitative results, odor descriptions and average peak area.

No	Compounds	CAS	RI (RT-5)	RI (RT-1701)	Odor description	Odor Threshold (mg/m3)	Peak area (mean ± SD)		
							SS-01	SS-02	SS-03
1	1-Butene	106–98-9	375	–	Aromatic	2 (air)	9,016 ± 230	1896 ± 49	3,530 ± 213
2	Isobutene	115–11-7	390	–	Coal gas	20 (air)	10,610 ± 271	2,503 ± 102	2,315 ± 197
3	Acetaldehyde	75–07-0	408	459	Aldehydic, Apple, Etheral, Floral, Fresh, Fruity, Pleasant, Pungent	9 × 10^−2^ (air)	13,489 ± 277	17,525 ± 263	22,156 ± 842
4	ethanol	64–17-5	438	591	Alcoholic, Ethanol, Etheral, Fragrant, Pleasant, Pungent, Strong, Sweet, Weak	2 × 10^2^ (air)	130,534 ± 1,649	12,484 ± 187	29,368 ± 930
5	Propenal	107–02-8	469	549	Acrid, Almond, Cherry, Choking, Hot fat, Pungent, Sharp, Sweet	0.1 (air)	16,228 ± 245	8,896 ± 195	9,009 ± 268
6	2-methylpropanal	78–84-2	522	634	Aldehydic, Baked potato, Burnt, Floral, Fresh, Fruity, Green, Malty, Pungent, Sharp, Spicy, Toasted	2 × 10^−2^ (air)	55,489 ± 513	nd	5,399 ± 160
7	butane-2,3-dione	431–03-8	601	696	Butter, Caramelized, Chlorine, Creamy, Fruity, Pineapple, Pungent, Spirit, Strong, Sweet	3 × 10^−3^ (air)	8,548 ± 1,183	nd	50 ± 100
8	3-methylbutanal	590–86-3	654	742	Aldehydic, Almond, Apple, Cheese, Chocolate, Fatty, Fruity, Green, Herbaceous, Malty, Peach, Toasted	2 × 10^−3^ (air)	26,962 ± 139	206 ± 120	3,129 ± 106
9	2-methylbutanal	96–17-3	664	748	Almond, Apple, Burnt, Burnt (strong), Choking, Cocoa, Coffee, Fermented, Fruity, Green, Iodoform, Malty, Musty, Nutty, Powerful, Sickly, Sour	3 × 10^−3^ (water)	47,485 ± 339	385 ± 154	3,911 ± 131
10	2,3-Pentanedione	600–14-6	700	793	Almond, Apple, Burnt, Butter, Butterscotch, Caramelized, Cheese, Creamy, Diacetyl, Fresh, Fruity, Grain, Malty, Nutty, Oily, Pungent, Sickly, Sweet	4 × 10^−2^ (air)	4,710 ± 225	285 ± 19	2,818 ± 158
11	ethyl isobutyrate	97–62-1	745	821	Alcoholic, Ethereal (sweet), Fruity, Fusel, Rubber, Strawberry, Sweet	3 × 10^−4^ (air)	1,616 ± 71	471 ± 21	868 ± 44
12	Pyridine	110–86-1	761	837	Amine, Burnt, Cold meat fat, Fishy, Nauseating, Pungent, Putrid, Rancid, Sharp, Solvent, Sour	0.3 (air)	932 ± 66	nd	nd
13	Hexanal	66–25-1	774	889	Acorn, Aldehydic, Fatty, Fishy, Fresh, Fruity, Grassy, Green, Herbaceous, Leafy, Sharp, Strong, Sweaty, Tallowy, Vinous	3 × 10^−2^ (air)	4,452 ± 156	893 ± 345	2,623 ± 94
14	2-hexanol	626–93-7	803	895	Cauliflower, Chemical, Fatty, Fruity, Terpenic, Winey	50 (air)	5,164 ± 57	947 ± 131	4,649 ± 266
15	ethyl 2-methylbutyrate	7,452-79-1	835	925	Apple, Blackberry, Cognac, Fruity, Green, Phenolic, Sharp, Strawberry, Sweet	2 × 10^−3^ (air)	6,854 ± 235	nd	nd
16	1-Hexanol	111–27-3	375	988	Alcoholic, Characteristic, Dry, Fatty, Floral, Fruity, Fusel, Grassy, Green, Hay, Herbaceous, Leafy, Oil, Pleasant, Resinous, Sharp, Sweet, Toasty, Woody (mild)	1 (air)	2,665 ± 43	1,635 ± 101	2,354 ± 50
17	Propyl butanoate	105–66–8	390	954	Fruity, Orange (moldy), Pineapple, Solvent	0.2 (air)	979 ± 28	46 ± 102	44 ± 88
18	Heptanal	111–71-7	408	1,007	Aldehydic, Citrus, Fatty, Fish (dry), Fresh, Fruity, Green, Heavy, Herbaceous, Oily, Ozone, Pesticide, Pungent, Putty, Rancid, Smoky, Solvent, Sweet	6 × 10^−2^ (air)	6,048 ± 120	nd	nd
19	2,5-dimethyl-3-furanthiol	55,764–23-3	438	1,043	Meaty, Sulfurous	9 × 10^−6^ (air)	921 ± 46	nd	416 ± 57
20	2-Ethyl-5-methylpyrazine	13,360–64-0	469	1,065	Coffee, Fruity, Grassy, Nutty, Pungent, Sweet	4 × 10^−2^ (air)	1,602 ± 168	nd	nd
21	γ-Terpinene	99–85-4	522	1,089	Citrus, Etheral, Fruity, Gasoline, Herbaceous, Lemon, Oily, Sweet, Terpenic, Turpentine, Woody	60 (air)	1,499 ± 151	571 ± 10	855 ± 26
22	nonan-2-one	821–55-6	601	1,206	Baked, Cheese, Earthy, Fatty, Fresh, Fruity, Green, Ketonic, Milk (hot), Mustard, Musty, Soapy, Spicy, Sweet, Varnish	0.9 (air)	592 ± 85	nd	nd

SD is standard deviation, peak area is presented as means ± SD (*n* = 5).

**Figure 8:**
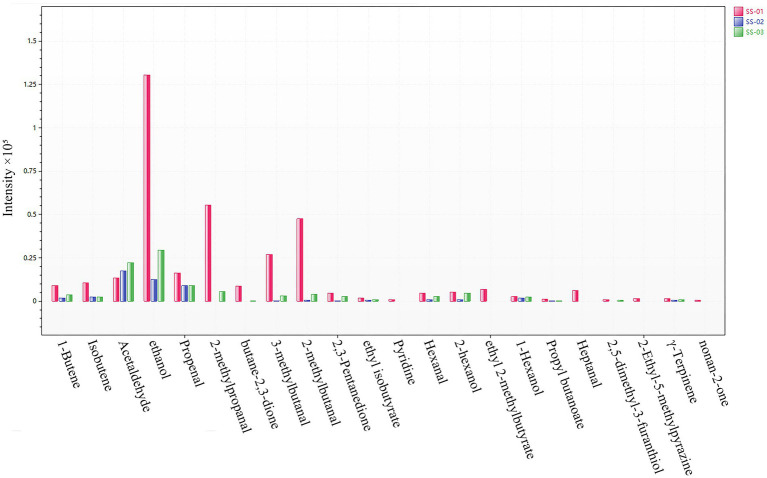
Histogram of differential compound contents.

In order to more intuitively compare the differences in the content of compounds in each sesame oil sample, a bar graph of the content of different compounds was drawn; in this graph, volatile compounds are used as the horizontal axis and the average peak area is used as the vertical axis, as shown in Figure 8. From the bar graph, it can be observed that the most obvious feature is SS-01, which is represented by red; and has the highest content of 21 compounds, including 1-Butene, Isobutene, ethanol, Propenal, 2-methylpropanal, butane-2,3-dione, 3-methylbutanal, 2-methylbutanal, and ethyl 2-methylbutyrate. Meanwhile, the content of Acetaldehyde is lower than that of the other two samples. The content of SS-03 is relatively stable, and the content of Acetaldehyde is higher than that of other samples. SS-02 did not detect any components with significantly higher content than in the other sesame oil samples, and the content of compounds was generally lower than that of other samples. The data in the figure show that the chemical composition of the volatile compounds in sesame oil is similar, but that the same chemical components of sesame oil treated via different processing methods are different; this indicates that there are differences in the quality of sesame oil when treated via different methods.

### 3.3 One-way ANOVA

**Figure 9:**
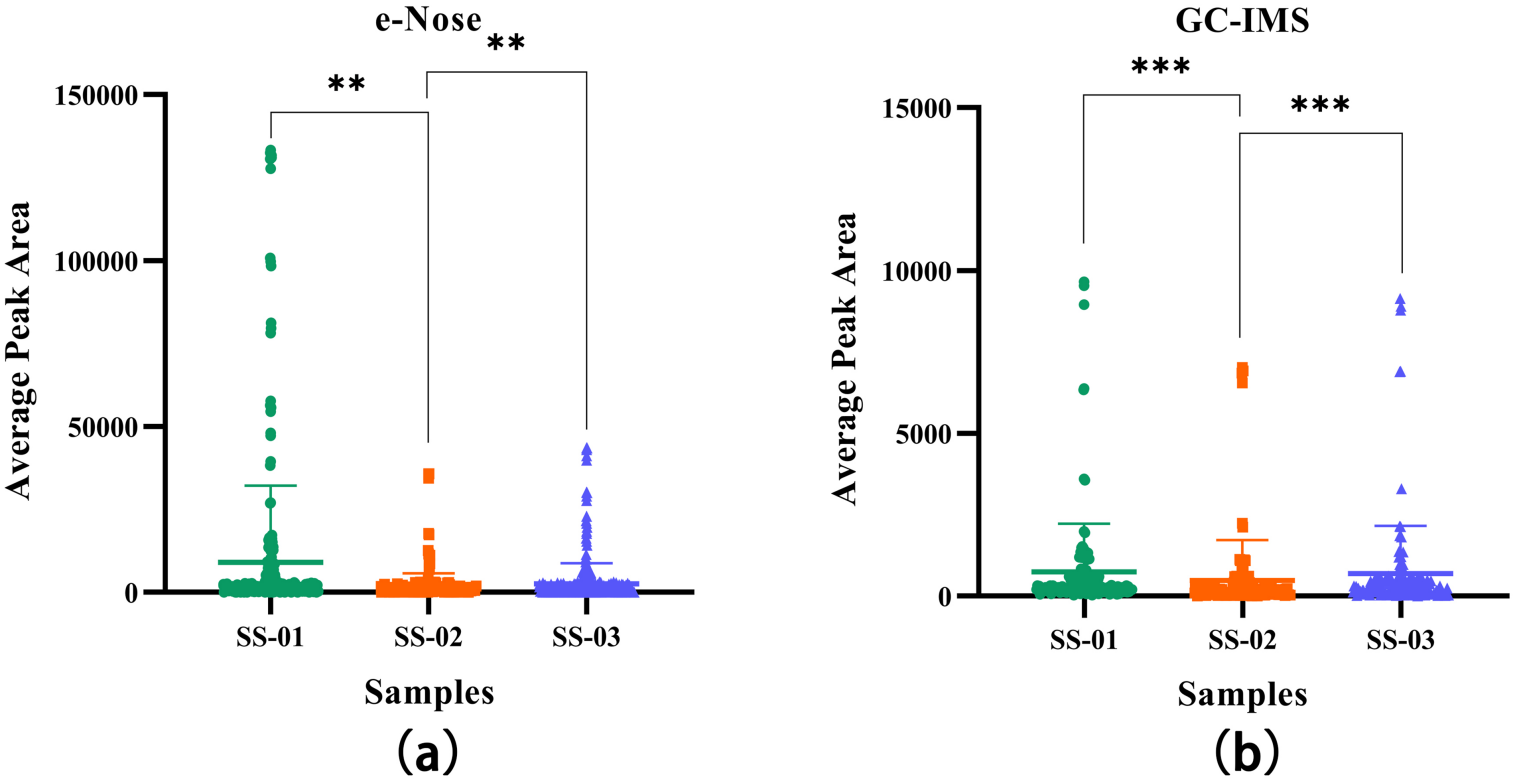
**(a)** One-way ANOVA results of VOCs in sesame oil processed by three different methods using e-Nose; **(b)** One-way ANOVA results of VOCs in sesame oil processed by three different methods using GC-IMS.

In order to determine whether different treatment methods have a significant impact on the VOCs of sesame oil, this study quantified the results through one-way ANOVA, providing more specific evidence for the conclusion. As shown in Figure 9, in the one-way ANOVA results of GC-IMS and e-nose, the VOCs of the SS-01 and SS-03 groups showed significant differences compared to the SS-02 group (*p* < 0.01).

## 4 Discussion

Sesame oil is an edible oil of high economic and nutritional value, widely used as a food seasoning and possessing a unique taste. Its aroma generally reflects its quality and influences consumers’ purchasing intentions. Most aroma compounds in oil are formed by various reactions during processing, including enzymatic and thermal reactions such as the Maillard reaction, Strecker degradation, caramelization, and lipid thermal reactions (21, 22).

In this study, the Heracles NEO ultra-fast gas-phase electronic nose and GC-IMS were utilized to detect differences in VOCs of sesame oil processed via different processing methods. Sensory evaluation was also performed. GC-IMS combined with chemometric analysis, objectively analyzed sesame oil odors and compared the effects of different processing methods on VOCs profiles. Moreover, one-way ANOVA was conducted on the volatile components detected by the two detection methods. The empirical results revealed substantial differences in the VOC profiles of sesame oil processed via different methods. A total of 60 VOCs were identified from the sesame oil samples via GC-IMS; including 16 aldehyde compounds (26.7%); 16 alcohol compounds (26.7%); 14 ketone compounds (23.3%); and 5 pyrazine compounds (8.3%). In addition, furans, terpenoids, thiazoles, pyrroles, esters and acids were identified.

The fingerprint and PCA results indicate that the VOCs obtained from the water substitution method are, primarily, ketones, thiazoles and pyrazine com-pounds. Ketones are mainly formed by beta oxidation of fatty acids, and con-tribute fatty flavor characteristics (23). 2,4,5-trimethylthiazole can be produced via L-cysteine, degradation and has a strong chocolate aroma and earthy smell, enhancing the flavor and taste (24). Pyrazines such as 2,3,5-trimethylpyrazine and 2-ethyl−5-methylpyrazine have a roasted or earthy smell, and are formed by the Maillard reaction; they have a low odor threshold and are used in the food industry (25, 26). The cold-pressing method results in a higher content of *γ*-Terpinene and 2-methyl-2-propenal. γ-Terpinene has the aroma characteristics of conifer and citrus fruits, and can significantly improve the flavor of products (27–29). 2-methyl−2-propenal plays an important role in the manufacture of spices and flavors (30). The hot-pressing method results in high contents of aldehydes such as 1-propanol, Hexanal, and (E)-2-Heptenal, which have aroma characteristics such as a fatty, green, fruity vanilla, and floral aromas (31). 2-Methyl-1-propanol compounds like 1-propanol have fresh, fruity and floral aromas and impact product aroma and taste. From the VIP results, it can be seen that 1-propanol-D, 1-hexanol-M, (E)-2-Heptenal-D, 2-Methyl-1-butanol-M, n-Pentanal-M, (E)-2-Heptenal-M, 3-Octanone and Heptaldehyde has the greatest impact on the flavor of sesame oil samples.

The Heracles NEO fast electronic nose results complemented the GC-IMS findings by providing a qualitative analysis of the VOC differences in sesame oil processed using different methods. The PCA results of the electronic nose show that the SS-01 sample is located alone on the right side of the area and is the sample with the largest odor difference. The positions of SS-02 and SS-03 are relatively close to each other, and the overall difference in odor between these two groups of samples is small. A total of 22 compounds were identified from the qualitative results of differential chromatographic peaks. The components of 1-Butene, Isobutene, Propenal, 2,3-Pentanedione, ethyl isobutyrate, Hexanal, 2-hexanol, and Propyl butanoate in SS-01 were higher than those of the other two groups. In addition, Pyridine, ethyl 2-methylbutyrate, Heptanal, and nonan-2-one were only in SS-01; ethanol and Acetaldehyde were only in SS-02; the Propenal component was higher than other components; the Acetaldehyde component was higher in SS-03 than the other two groups; and ethanol, Acetaldehyde and Propenal were higher than other components. From the histogram showing the differential compound content, it can be inferred that ethanol, heptanal, ethyl 2-methylbutyrate, γ-Terpinene, Pyridine, and 2-Ethyl-5-methylpyrazine constitute the main flavor characteristics of sesame oil. Aligning with previous studies on the aroma compounds in sesame oil, when evaluating the flavor characteristics, Fruity, Sweet, and Pungent were the main flavor characteristics. Heptanal and ethyl 2-methylbutyrateju both presented Fruity, Green and Sweet flavors, which were only reflected in SS-01. Acetaldehyde presented Aldehydic, Apple, Fruity and Pungent flavors, and was found in higher amounts in SS-02 and SS-03.

Huang et al. used scanning electron microscopy (SEM) to observe the microstructure of sesame and studied the effects of different heat treatment methods on the processing quality of sesame and cold pressed oil (8). Rahmania et al. used high-performance liquid chromatography tandem mass spectrometry (LC–MS/MS) to investigate the stability of sesame oil (32). However, this study innovatively used Heracles Neo ultrafast gas-phase electronic nose combined with GC-IMS technology for chemometric analysis, and systematically compared the differential effects of water substitution, cold pressing, and hot pressing on the volatile organic compounds and active aroma components of sesame oil for the first time. This achievement provides a scientific basis for the optimization of sesame oil processing technology at the level of volatile components, fills the research gap in the impact mechanism of different processes on sesame oil flavor compounds, and has important guiding significance for promoting the technological upgrading and quality improvement of sesame oil industry.

Compared with GC–MS, the combination of GC-IMS and Heracles NEO ultra-fast gas-phase electronic nose has the following advantages:

Higher sensitivity: GC-IMS can detect volatile organic compounds (VOCs) at ppb, making it suitable for detecting low-concentration volatile substances (33–35); faster analysis speed: GC-IMS does not require sample enrichment or concentration, significantly reducing analysis time (33, 36). Heracles NEO, with its rapid gas chromatography technology and automatic sampler, can analyze up to 200 samples per day, achieving high-throughput detection (37); lower cost: GC-IMS does not require a vacuum system, has a short start-up stabilization time, and uses renewable gasses (such as nitrogen) instead of non-renewable helium, reducing operating costs (36). Heracles NEO also reduces costs by minimizing solvent use and simplifying sample preparation (37); better compound separation: GC-IMS achieves double separation of complex mixtures through gas chromatography for preliminary separation and ion mobility spectrometry for secondary separation (34). This improves the separation of VOCs and avoids the ambiguity of direct detection of mixed gasses by electronic nose technology, enhancing detection accuracy.

The cold-pressing method involves low-intensity heat treatment and is classified as a physical pressing process with negligible chemical changes, producing distinctive flavor compounds such as hydrocarbons, aldehydes, and alcohols (38). In contrast, the hot-pressing method employs high-intensity heat treatment, which can induce Maillard reactions and thermal degradation of fats, resulting in the formation of characteristic flavor compounds such as pyrazines and phenols (39, 40). The water substitution method, characterized by moderate heat treatment, also triggers Maillard reactions among other processes, thereby generating key flavor compounds including pyrazines, phenols, aldehydes, and esters (21). Specifically, cold pressing preserves natural components at low temperatures, yielding a mild flavor profile; hot pressing, through high-temperature activation, produces a rich yet less stable flavor; and the water substitution process employs a gentle treatment that facilitates the formation of complex flavor compounds via water-mediated mechanisms. Essentially, the differences among these three techniques stem from the varying intensities of heat treatment and processing pathways, which influence the degradation reactions of proteins, fats, and sugars present in sesame.

This exploratory study evaluates the effects of various processing methods on the VOCs in sesame oil samples from a single origin, which may limit the generalizability of the findings. Moreover, constraints related to experimental conditions and time resulted in a relatively small sample size, potentially increasing the variability in the detection of volatile components. Future research should address these limitations by expanding the diversity of sample sources and incorporating multiple control experiments. In future research, we will further investigate how factors such as temperature, enzyme activity, lipid oxidation, or the Maillard reaction affect the production of specific volatile compounds.

## 5 Conclusion

A total of 74 VOCs were detected in the three sesame oil samples, which were from GC-IMS and Heracles NEO ultra-fast gas-phase electronic nose (60 VOCs were detected via GC-IMS, 22 VOCs were detected via GC-IMS, among them, 8 VOCs were simultaneously detected via GC-IMS and Heracles NEO ultra-fast gas-phase electronic nose). The sesame oil produced via the water substitution method was rich in more than 42 VOCs, including Cyclopentanone, 1-Pentanol and had a more unique and richer flavor; the sesame oil produced via the cold-pressing method contains 4 VOCs, for example, *γ* -terpinene with an original fruity flavor; and the sesame oil processed by the hot-pressing method was rich in 29 VOCs, including 2-methyl-1-propanol, and had a better fat aroma. In this study, a comprehensive analysis of flavor changes during sesame oil processing was performed through a combination of electronic nose and GC-IMS technology and chemometric methods, and key flavor markers were identified. It can not only strengthen the scientific evaluation of food quality and safety, but also promote the development of the food industry in a more logical and specific direction. Through the integration of these advanced technologies, food producers and regulators can determine the status of food with unprecedented accuracy, ensuring that consumers enjoy a safe, high-quality, and expected food experience. This study helps to improve the quality and flavor of sesame oil from the perspective of volatile components, facilitating technological innovation and industrial upgrades.

## Data Availability

The original contributions presented in the study are included in the article/supplementary material, further inquiries can be directed to the corresponding author/s.
